# Rift Valley Fever in Chad

**DOI:** 10.3201/eid1005.030621

**Published:** 2004-05

**Authors:** David Ringot, Jean-Paul Durand, Hughes Tolou, Jean-Paul Boutin, Bernard Davoust

**Affiliations:** * Interarmy Veterinary Sector for Montpellier, Nîmes, France; † Tropical Medicine Institute of the French Defense Medical Service, Marseille, France; ‡ Medical Service Directorate for Lyon, Lyon, France

**Keywords:** Rift valley fever, Zoonotic hemorrhagic fever, Arboviruses, Central Africa, Chad

## Abstract

To evaluate the importance of human exposure to Rift Valley fever virus in Chad, investigations were carried out to determine specific antibody prevalence in domestic ruminants during the 2002 rainy season. Results highlighted recent, substantial, active transmission of this virus.

The virology laboratory of the unit of the Tropical Medicine Institute of the French Defense Medical Service (IMTSSA) investigated some cases of self-limiting nonmalarious febrile syndromes occurring among soldiers stationed in Chad. By using C6/36 and Vero cell lines, peripheral blood lymphocytes collected from two soldiers on duty in Chad during the 2001 rainy season were cocultured. Two strains of Rift Valley fever virus (RVFV) were isolated and identified by using indirect immunofluorescence, reverse transcriptase–polymerase chain reaction, and sequencing (*1*,*2*). To determine the potential for human exposure to RVFV, a seroprevalence investigation evaluated antibody prevalence in sheep, goats, and horned cattle among animals taken to slaughterhouses of N’Djamena and Abéché during the 2002 rainy season.

RVFV is a member of the genus *Phlebovirus*, family B*unyaviridae*, and was first isolated in Kenya in 1930. Transmitted by a wide variety of mosquitoes from several genera, the virus may cause abortion in pregnant livestock and high death rate in young animals. RVFV has caused influenzalike disease in humans, and it occasionally leads to more serious complications, such as retinitis, meningoencephalitis, or severe hemorrhagic fever with a high death rate.

In Chad, RVFV has never been officially recognized by either the World Health Organization or by the International Office of Epizooties. Nevertheless, three facts suggest that the virus is present in Chad’s animal population. First, RVFV is generally thought to exist in the enzootic state in Central Africa in sheep and wild animals (*3*). Second, a study undertaken by the Pasteur Institute of Paris in collaboration with the EMVT (Département Elevage et Médecine Vétérinaire Tropicale du Centre de Coopération Internationale pour la Recherche en Agronomie pour le Développement) showed that 4% of sheep bred in Chad and Ethiopia had anti-RVFV neutralizing antibodies (*4*). Third, RVFV was identified in Sudan, Niger (*5*), and Nigeria, countries that border Chad.

## The Study

During the 2002 rainy season (August through October), within the slaughterhouses of the cities of N’Djamena (southwestern Chad between the 10th and 15th parallels, a few kilometers from Cameroon) and Abéché (220 km east of N’Djamena), blood samples were collected from randomly selected sheep, goats, and horned cattle (Table 1). According to the veterinary services of Chad’s Ministry for Breeding, these animals were born and bred in Chad. Furthermore, they were gathered in parks a maximum of 3 days before slaughter. Sites were selected for their proximity to an area where French troops were deployed and also because the N’Djamena slaughterhouse, in particular, receives cattle from various parts of Chad.

**Table 1:** Number of blood samples per species and site

Animal	N’Djamena slaughterhouse	Abéché slaughterhouse	Total
Sheep	211	89	300
Goats	102	37	139
Horned cattle	99	15	114
Total	412	141	553

Each sample was accompanied by information on the age of the animal (teeth examination), species, sex, and origin. Blood was centrifuged within 24 hours of collection. The serum was transferred into cryotubes and frozen at –80°C so that samples would arrive at IMTSSA for analysis still frozen (–20°C). Each sample was systematically tested for RVFV-specific immunoglobulin (Ig) G by using an enzyme-linked immunosorbent assay (ELISA). First, ELISA screening was done by using antigen capture (by mouse hyperimmune ascitic fluid) and detecting specific IgG in the diluted serum (1/500). The antigen used was a precipitate (polyethylene glycol 6000) of the supernatant of Vero cells infected with the RVFV clone 13. (This strain was isolated from a person in the Central African Republic [*6*] and is probably less dangerous than other strains for laboratory workers).

On the same ELISA plate as negative antigen, the serum was tested with Dugbe, a non-cross-reactive Nairovirus. All IgG-positive serum samples were retested for IgG (with a negative, noninfected Vero antigen) and IgM by using the M-antibody capture method. The most frequently used techniques for detecting anti-RVFV antibodies are immunofluorescence, plaque reduction neutralization assay, and immunoenzymatic assays (*7*). Because RVFV cross-reacts with many other phleboviruses (*5*), the choice of techniques used for this study was influenced by their sensitivity and specificity. Seroneutralization is described as the reference method for specificity (no cross-reaction with other phleboviruses) (*7*), but the need for cell culture makes it unsuitable for screening large numbers of serum samples (*8*). ELISA was preferred, since it is considered an efficient alternative in terms of sensitivity, specificity, and ease of use (*7*,*8*).

Serum samples were considered positive when the ratio between optical density associated with RVFV antigen and that associated with the Dugbe antigen was >3.5. Serum specimens demonstrating anti-RVFV IgG were validated in parallel by immunotransfer (Western blot) with a high threshold of positivity. Only serum samples containing both specific antibodies against the envelope glycoproteins (G1 and G2) and the nucleocapsid (NC) protein were considered positive. Comparative results between the two techniques confirm high specificity of ELISA (97% of the serum samples positive by ELISA were confirmed by Western blot). This high specificity has been described previously by crosschecking results with those of virus neutralization assay (*8*).

## Conclusions

The relatively high prevalence of RVFV (Table 2) combined with the fact that 41% of IgG-positive animals are also IgM-positive (in cattle, these antibodies appear on the fourth day (*9*) after natural infection and persist for 2–6 months (*10*) underscore the seriousness of the situation in Chad. Indeed, many articles describe how domestic ruminants are an early and sensitive indicator of human epidemics (*7*) and how outbreaks of human infection are preceded by amplification cycles among animals (*7*).

**Table 2:** Results of the cross-sectional investigation of Rift Valley fever antibody prevalence among sheep, goats, and horned cattle, Chad, 2002 rainy season^a^

Animal	Average age (y)	IgG+^b^ animals (%)	Average age of IgG+ animals (y)	IgG+ animals age <1 y (% of those age <1 y)	% of animals killed in an area that were IgG+	Sex of IgG+ animals	IgG+ animals confirmed by WB	IgM+ animals (% IgG+)
Sheep	2.3	32/300 (10.7)	1.8	8 (12)	14.8% N, 1.1% A	7.5% of M, 12.8% of F	31	16 (53.3)
Goats	1.7	12/139 (8)	2.2	4 (6)	9% N, 5% A	6.7% of M, 10.8% of F	NA	4 (33.3)
Horned cattle	7.5	5/114 (4)	6.2	NA	5% N, 0% A	0% of M, 4.7% of F	NA	NA
Total	3.2	49/553 (8)	2.8	14 (10.5)	11% N, 2% A	4.4% of M, 11.4% of F	NA	20 (45.4)

aIg, immunoglobulin; WB, Western blot; N, N’Djamena; A, Abéché; NA, not available. bSerum positivity was established when the ratio between the optical density of the Rift Valley fever virus antigen and that of the Dugbe antigen was >3.5.

The 1987 epizootic-epidemic in Mauritania was predicted by the Pasteur Institute of Dakar; by using a seroepidemiologic study among domestic animals, researchers showed that the virus had been circulating for at least 6 months in animal hosts and that an amplification cycle of the disease was in progress (*11*). We fear that, as occurred in Burkina Faso in 1987 (*1*), ecologic changes or climatic conditions favorable to vector proficiency (e.g., periods of intense rain associated with epizootic appearance in Kenya [*12*] and South Africa [*13*]) can increase, in areas where the virus circulates, antibody prevalence in animals and can lead to human cases (*4*). This risk appears even more important since human outbreaks are specifically preceded by an increase of antibody prevalence among animal populations. A study by the Pasteur institute of Paris showed that 4% of sheep bred in Chad and Ethiopia had anti RVFV antibodies (*14*), and these figures were repeatedly confirmed (*4*).

Data regarding origin (source and path) could not be collected for animals led to the N’Djamena slaughterhouse (all that was known was that they were born and bred in Chad) and are imprecise for those received at the Abéché slaughterhouse (local source not specified). Thus, charting the distribution of RVFV-positive animals and the geographic distribution of the virus is not possible. Nevertheless, the weak antibody prevalence in animals killed in the Abéché slaughterhouse should be noted, which allows us to conclude that this particular area is still isolated from RVFV.
